# Stabilization, Characterization, and Selective Removal of Cystatin C Amyloid Oligomers*

**DOI:** 10.1074/jbc.M113.469593

**Published:** 2013-04-29

**Authors:** Gustav Östner, Veronica Lindström, Per Hjort Christensen, Maciej Kozak, Magnus Abrahamson, Anders Grubb

**Affiliations:** From the ‡ Department of Clinical Chemistry, Lund University Hospital, S-22185 Lund, Sweden,; the § Dako Denmark A/S, DK-2600 Glostrup, Denmark, and; the ¶ Department of Macromolecular Physics, Adam Mickiewicz University in Poznań, 61-614 Poznań, Poland

**Keywords:** Amyloid, Protease Inhibitor, Protein Aggregation, Protein Conformation, Protein Domains, Protein Misfolding, Cystatin C, Domain Swapping

## Abstract

The pathophysiological process in amyloid disorders usually involves the transformation of a functional monomeric protein via potentially toxic oligomers into amyloid fibrils. The structure and properties of the intermediary oligomers have been difficult to study due to their instability and dynamic equilibrium with smaller and larger species. In hereditary cystatin C amyloid angiopathy, a cystatin C variant is deposited in arterial walls and cause brain hemorrhage in young adults. In the present investigation, we use redox experiments of monomeric cystatin C, stabilized against domain swapping by an intramolecular disulfide bond, to generate stable oligomers (dimers, trimers, tetramers, decamers, and high molecular weight oligomers). These oligomers were characterized concerning size by gel filtration, polyacrylamide gel electrophoresis, and mass spectrometry, shape by electron and atomic force microscopy, and, function by assays of their capacity to inhibit proteases. The results showed the oligomers to be highly ordered, domain-swapped assemblies of cystatin C and that the oligomers could not build larger oligomers, or fibrils, without domain swapping. The stabilized oligomers were used to induce antibody formation in rabbits. After immunosorption, using immobilized monomeric cystatin C, and elution from columns with immobilized cystatin C oligomers, oligomer-specific antibodies were obtained. These could be used to selectively remove cystatin C dimers from biological fluids containing both dimers and monomers.

## Teaser

**Background:** Protein oligomers may be toxic, but are unstable, difficult to study, and challenging therapeutic targets.

**Results:** Oligomers can be stabilized and used to generate and isolate antibodies with high selectivity for amyloid oligomers.

**Conclusion:** Oligomer stabilization is a powerful strategy to study and target amyloid oligomers.

**Significance:** We describe methods to generate oligomer-specific antibodies for molecular characterization, diagnosis and treatment of amyloid disorders.

## Introduction

A major challenge in future healthcare is managing the growing number of patients affected by protein conformational disorders. Protein misfolding is implicated in the pathogenesis of neurodegenerative pathologies like Alzheimer, Huntington, and Parkinson diseases, the spongiform encephalopathies, age-related diabetes mellitus, cystatin C amyloidosis with brain hemorrhage, and a number of other conditions characterized by tissue amyloid deposition. To date, at least 27 different amyloidogenic proteins involved in human disease have been identified (1).

The molecular pathophysiological process in the amyloid disorders usually involves the transformation of a soluble functional monomeric protein into potentially toxic aggregates/oligomers and insoluble amyloid fibrils (2, 3). Several forms of molecular aggregates of the amyloidogenic proteins are known to occur during transition of the monomeric protein into amyloid fibrils (3, 4). However, the structure and properties of the intermediary oligomers have been difficult to study due to their instability and dynamic equilibrium with smaller and larger species. This has hampered the development of a deeper understanding of the molecular pathophysiology of the disorders and strategies for their treatment (5, 6).

We have previously shown that a stabilization of the monomeric form of cystatin C by insertion of a disulfide bridge will stop its transition into amyloid fibrils by preventing domain swapping of the monomer (7). In the present work we use redox experiments involving the disulfide-stabilized cystatin C monomer to produce stable intermediary oligomers of cystatin C. This allows characterization of the oligomers as well as development of a strategy to selectively remove oligomers from biological fluids containing both oligomers and monomers of the protein.

## EXPERIMENTAL PROCEDURES

### 

#### 

##### Materials

Chromatography columns and resins, gel filtration protein standards, and the ÄKTA FPLC system were obtained from GE Healthcare. Analytical gel filtrations were performed on Superdex 75 PC 3.2/30 or Superdex 200 PC 3.2/30 columns in a HPLC system, including a 600e controller and a 2996 Photodiode Array detector from Waters (Sollentuna, Sweden). Fluorimeter (Fluoroskan Ascent), spin desalt columns, dialysis cassettes (Pierce, 10,000 molecular weight cut-off (MWCO)),^2^ and membrane tubings (Spectrapore, 10,000 MWCO) were from Fisher Scientific. GrantBio Thermoshaker was obtained from Grant Instruments (Hillsborough, NJ). SpectraMax 340PC^384^ microplate absorbance reader was from Molecular Devices (Berkshire, UK). Ultrafiltration devices (Sartorius Vivaspin 20, 5,000 MWCO) were from A-filter (Mölnlycke, Sweden). Microtiter plates (384w number 264340, 96w MaxiSorp) and polyolefin sealing tapes were from Nunc (Roskilde, Denmark). Polypropylene tubes, 0.5 and 1.5 ml, were purchased from Applied Biosystems and VWR (Stockholm, Sweden), respectively. Nitrocellulose membranes were obtained from Ancos (Højby, Denmark). NuPAGE polyacrylamide gels, sample loading buffer, Mark12-unstained standards, phosphate-buffered saline (PBS, Invitrogen tablets), and polyclonal rabbit anti-oligomer antibodies (A11) were obtained from Invitrogen. Silver staining kit, ChemiDoc XRS+ system, and ImageLab software (2011, version 4.0) were purchased from Bio-Rad. Dried nonfat milk was from Semper (Sundbyberg, Sweden). Polyclonal rabbit anti-human cystatin C antibodies, horseradish peroxidase (HRP)-labeled polyclonal swine antibodies against rabbit immunoglobulins and HRP-labeled polyclonal rabbit antibodies against mouse immunoglobulins were from Dako (Glostrup, Denmark). Immobilon Western enhanced chemiluminescence (ECL) substrate was from Millipore (Billerica, MA). Papain and legumain substrates were acquired from Bachem Feinchemikalien (Bubendorf, Switzerland). Agarose (SeaKem LE) was purchased from BioWhittaker Molecular Applications (Rockland, ME). Coomassie Brilliant Blue R-250 was from VWR. Brij-35 was obtained from Kebo (Stockholm, Sweden). Guanidine hydrochloride (GdnHCl, 8 m in H_2_O, number G9284) was from Sigma. All other reagents were of at least reagent grade and, unless specified, obtained from Sigma.

##### Protein Production and Purification

Protocols for recombinant expression and purification of all cystatin C variants used in this work have been described earlier (7–9). In short, human wild type (wt) cystatin C and double cysteine variant (stab1) cystatin C were expressed and purified from the soluble fractions of periplasmic *Escherichia coli* extracts using ion-exchange and size exclusion chromatography (SEC), subsequently stored as lyophilized powder at room temperature, and reconstituted in appropriate buffers just before use. The L68Q cystatin C variant was purified from solubilized *E. coli* inclusion bodies as previously described (9), with the addition of a buffer exchange refolding step using HiTrap Desalting 5-ml columns equilibrated in the SEC running buffer, prior to purification by SEC. Both monomers and dimers of L68Q cystatin C could be isolated by SEC. Mammalian legumain (EC 3.4.22.34) was prepared from pig kidneys and purified to partial homogeneity as described by Chen *et al.* (10) with an additional heparin column chromatography step (11). Papain (EC 3.4.22.2) was purchased from Sigma (number P4762).

##### Protein Quantification

Protein concentrations of recombinant cystatin C variants were measured by UV absorption spectroscopy at 280 nm in a NanoDrop 2000 spectrophotometer (Fisher Scientific) using a mass extinction coefficient (*A*_280,__1 cm_^0.1%^) of 0.83 (12). The same method was used for determination of the concentration of cystatin C oligomers and, for these samples, mass concentrations are given throughout the report (in units of mg/ml or mg/liter). A mass extinction coefficient (*A*_280,__1 cm_^0.1%^) of 1.35 was assumed for purified rabbit immunoglobulins (13). Plasma cystatin C was measured either by an automated particle-enhanced immunoturbidimetric assay using a Roche-Hitachi Cobas 6000 analysis platform and a calibrator of isolated recombinant human cystatin C (14) or, in the case of fractionated plasma, by a sandwich enzyme-linked immunosorbent assay (ELISA) previously described (15).

##### Optimization of Conditions for Oligomer Formation

Lyophilized stab1 cystatin C was dissolved in PBS and aliquots of the stock solution (1 mg/ml) were added to solutions of GdnHCl in PBS, giving final concentrations of GdnHCl varying from 0 to 1.0 m and a final cystatin C concentration of 0.2 mg/ml (15 μm). Portions of 10 μl of the mixtures were distributed into the 25-μl wells of 384-well microtiter plates and either of the reducing agents dithiothreitol (DTT) or tris(2-carboxyethyl)phosphine was added to each well to final concentrations (10.4 μl of total assay volume) ranging from 0 to 150 μm (*i.e.* up to 10-fold molar excess of the reducing agents). The plates were sealed with sealing tapes and incubated at 37 or 40 °C. For each time point (1, 4, or 24 h) the solutions were analyzed by agarose gel electrophoresis, as described previously (16), with up to 42 samples per gel. Dimerization of cystatin C results in an anodal mobility shift so that a mixture of monomeric and dimeric cystatin C results in two protein bands after electrophoretic separation under native conditions in an agarose gel (17). The optimal conditions for the induction of larger oligomers (trimers, *etc.*) were established in 10.4-μl assay volumes by increasing the cystatin C concentration to 3 mg/ml under the optimal dimer-inducing conditions, *i.e.* equimolar DTT and 1 m GdnHCl, and decreasing the incubation temperature and time, with the results followed by SEC. The incubation mixtures were transferred to 0.5-ml tubes and centrifuged at 10,000 × *g* for 10 min and 5 μl were analyzed in a HPLC system using a Superdex 75 PC 3.2/30 column run in 150 mm ammonium bicarbonate, pH 7.8, with a flow rate of 0.1 ml/min and the eluate monitored by its absorption at 280 nm. In scale-up experiments of oligomer-inducing mixtures, using the optimized conditions, 0.5-ml incubation volumes showed identical chromatograms compared with 10.4-μl volumes.

##### Purification of Stabilized Oligomers

Lyophilized stab1 cystatin C was reconstituted in 0.5 ml of PBS in 1.5-ml tubes, the concentration was measured in 1.5-μl droplets using a NanoDrop 2000 spectrophotometer, and concentrations were adjusted before the addition of GdnHCl from a 8 m stock solution, giving final concentrations of GdnHCl of 1 m. To this mixture was added freshly dissolved DTT in PBS at 0.9 mg/ml (6.25 mm) giving final concentrations of DTT and cystatin C of 225 μm (corresponding to a cystatin C concentration of 3 mg/ml), and the tubes were kept at room temperature for 2 h. After centrifugation at 10,000 × *g* for 10 min, 0.5-ml samples were injected in an ÄKTA FPLC system running a Superdex 75 GL 10/300 column in 150 mm ammonium bicarbonate, pH 7.8, with a flow rate of 0.5 ml/min and the eluate was monitored by its absorption at 280 nm. Fractions of 0.5 ml were collected and the tubes stored open to ambient air overnight at 4 °C to allow re-formation of disulfide bonds by spontaneous oxidation. Fractions were analyzed by nonreducing silver-stained SDS-PAGE as described below, pooled, and concentrated to 1–3 mg/ml in ultrafiltration devices (5,000 MWCO). The protein concentration was measured, samples were aliquoted and either lyophilized or stored at 4 or −20 °C.

##### SDS-PAGE

Purified monomers/oligomers were analyzed by SDS-PAGE using pre-cast NuPAGE BisTris gradient gels (4–12%). Samples were boiled, without the addition of reducing agents, in 2% SDS loading buffer according to the manufacturer and the gels run with the MES buffer system, at 200 V for 35 min. The gels were either stained with Coomassie or silver-stained as described by the manufacturer. The gels were digitized in a ChemiDoc system and analyzed using the included ImageLab software.

##### Mass Spectrometry

Isolated monomers and oligomers were analyzed by mass spectrometry using a matrix-assisted laser desorption/ionization time-of-flight (MALDI-TOF) ultrafleXtreme mass spectrometer (Bruker Daltonics, Bremen, Germany). Samples at 1 mg/ml in 150 mm ammonium bicarbonate, pH 7.8 (nonreducing buffer), were mixed 1:1 with the matrix 2,6-dihydroxyacetophenone and 1 μl was applied to the ground steel plate before analysis by a positive method operating in reflectron mode and a 25 kV acceleration voltage. The analyses were calibrated using external calibrants (Bruker Daltonics) and re-calibrated using wt cystatin C (*M*_r_ 13,343).

##### Analytical Gel Filtration

Eight-μl samples of purified monomers/oligomers were centrifuged at 10,000 × *g* for 10 min and analyzed in a HPLC system using a Superdex 200 PC 3.2/30 column equilibrated in 150 mm ammonium bicarbonate, pH 7.8, with a flow rate of 0.1 ml/min and the eluate was monitored by its absorption at 280 nm. The column was calibrated with protein standards (ribonuclease, *M*_r_ 13,700; chymotrypsinogen, *M*_r_ 25,000; ovalbumin, *M*_r_ 44,000; BSA, *M*_r_ 67,000; aldolase, *M*_r_ 158,000; ferritin, *M*_r_ 440,000). A calibration curve was constructed from calibrator protein elution volume peaks and the mass of each cystatin C oligomer calculated from its elution volume.

##### Transmission Electron Microscopy

Protein samples were diluted 1:10 or 1:40 in 150 mm ammonium bicarbonate, pH 7.8, and 5 μl were applied to glow-discharged carbon-coated copper grids (400 mesh) and after 1 min of adsorption the excess liquid was removed with filter paper. The samples were negatively stained with 5 μl of 2% (w/v) aqueous uranyl acetate for 30 s before the staining solution was removed with filter paper. The samples were examined with a Technai G^2^ Spirit electron microscope (FEI, Eindhoven, The Netherlands), operating at an excitation voltage of 80 kV and equipped with an Eagle 16 megapixel CCD camera. Electron micrographs were analyzed using the software ImageJ (18).

##### Atomic Force Microscopy

Atomic force microscopy (AFM) measurements were carried out using the NanoWizard II system (JPK Instruments, Berlin, Germany). Oligomer samples were prepared as described above and diluted 1:2000 using double-distilled H_2_O. The sample solutions (5 μl) were deposited on a freshly cleaved mica surface and dried before visualization. The intermittent (air) contact mode imaging was carried out with a silicon nitride cantilever. The images were analyzed using the software package Gwyddion (2012, version 2.30) (19) and the diameter of the objects observed was measured in cross-sectional analyses at two-thirds of full height.

##### Enzyme Inhibition Assay

The inhibitory capacity of purified and stabilized cystatin C oligomers was measured using the cysteine proteases papain and legumain. When setting up the assays (20), enzymes and fluorogenic substrates were titrated to give linear values of substrate development as measured in 100-μl assay volumes in a plate-type fluorometer at 37 °C during 30 min. Papain was prepared by dissolving lyophilized powder (Sigma) in Milli-Q water to 1 mg/ml and mixing 20 μl of a 1:5,000 dilution (in 0.01% (w/v) Brij-35) into the reaction mixtures containing 55 μl of assay buffer (400 mm sodium phosphate, pH 6.5, 4 μm DTT, 4 mm EDTA). Five-μl samples of purified stab1 monomers/oligomers or monomeric wt cystatin C at equal mass concentrations in 150 mm ammonium bicarbonate, pH 7.8, were added to the papain solution and the mixtures preincubated for 10 min at 37 °C. Twenty microliters of the synthetic peptide substrate *Z*-Phe-Arg-NHMec (from a 200 μm stock solution in 0.01% (w/v) Brij-35) were added and fluorescence was measured every 30 s at excitation and emission wavelengths of 355 and 460 nm, respectively. All measurements were carried out in duplicates. Full enzymatic activity (Fig. 4*A*) was defined as the mean of fluorescence values obtained after a 20-min substrate development in mixtures without the addition of monomeric, or oligomeric, cystatin C. Essentially the same protocol was employed in legumain inhibition assays using the fluorogenic substrate *Z*-Ala-Ala-Asn-AMC at 20 μm and assay buffer composed of 100 mm sodium citrate, pH 5.8, 4 μm DTT, 1 mm EDTA, and 0.1% (w/v) CHAPS (final concentrations).

##### High Molecular Weight (HMW)-Oligomer and Fibril Formation Assay

Wild type cystatin C was reconstituted in 50 mm sodium acetate, pH 4.0, 100 mm NaCl, and the protein concentration adjusted to 1 mg/ml. Purified stab1 cystatin C monomers and oligomers were subjected to buffer exchange into the sodium acetate buffer using spin columns (0.5 ml) with desalting resins according to the manufacturer's instructions and the protein concentration was adjusted to 1 mg/ml. Ten-μl samples were distributed into the wells of 384-well microtiter plates and the plates placed in a thermoshaker operating at 48 °C and at a speed of 900 rpm in orbits of 2 mm. HMW-oligomers/fibrils were visualized by electron microscopy as described above, or nonreducing SDS-PAGE using silver-stained NuPAGE gels as described above, but without boiling samples and with a low (0.1% (w/v)) SDS sample buffer, according to Ref. 21.

##### Rabbit Immunization

Immunizations were performed according to the ethical requirements and in-house protocols at Dako Denmark A/S. Prepared and isolated as described above, the stabilized dimers and HMW-oligomers, respectively, were used as immunogens in two groups of rabbits. Each group received initial and repeated booster injections of 0.1 mg of dimeric or 0.05 mg of HMW-oligomeric cystatin C. The immunoglobulin fractions of the antisera were isolated using standard ammonium sulfate precipitation and protein A-Sepharose fractionation, according to the instructions of the manufacturer. The immunoglobulins were stored in neutral buffer (20 mm Tris, pH 7.4, 150 mm NaCl) at −20 °C. The specific reactivity of antisera and immunoglobulin fractions against monomeric, dimeric, and HMW-oligomeric cystatin C was tested by Ouchterlony radial immunodiffusion as described below.

##### Affinity Purification of Oligomer-specific Antibodies

To remove monomer-binding antibodies from the oligomer-reactive immunoglobulin fractions, a two-step purification procedure, illustrated in Fig. 7, using an ÄKTA FPLC, was employed. A 20-mg batch of monomeric wt cystatin C was immobilized on HiTrap NHS-activated Sepharose (5 ml column), according to the instructions of the manufacturer. The immunoglobulin fractions were applied to the monomer-coupled column and flow-through fractions with significant absorption at 280 nm were pooled and concentrated by ultrafiltration (5,000 MWCO), before use in the second step. In the second purification step, a 2-mg batch of isolated HMW-oligomers was coupled to HiTrap NHS-activated Sepharose (1 ml column) and the flow-through fractions from the first column were applied to the oligomer-coupled resin. The oligomer-specific antibodies were eluted from the column with acidic buffers (100 mm glycine, pH 3.0, 0.5 m NaCl), dialyzed against PBS, and stored at −20 °C.

##### Double Radial Immunodiffusion

The Ouchterlony procedure was used for in-gel immunodiffusion assays (22).

##### Agarose Gel Electrophoresis to Test the Selectivity of Oligomer-specific Antibodies in Free Solution

Agarose gel electrophoresis was preformed as described (23). Antibodies and antigens (*i.e.* stabilized monomers and dimers, at equal final concentrations) were mixed in 10-μl volumes in wells of 384-well plates and allowed to react at room temperature for 30 min. Ten-μl samples were applied to the gel and electrophoresed at 250 V for 30 min at 4 °C. Gels were stained with Coomassie and band patterns were digitized by optical scanning in an EPSON 1680 Pro flatbed scanner. Protein band patterns were subjected to densitometric analyses using ImageJ software.

##### Immunoblotting

The dot blot procedures were performed according to the protocol from the manufacturer of the A11 antibodies. Briefly, 5-μl protein samples were applied to nitrocellulose membranes and allowed to dry. For A11 dot blots, the sample concentration was 1 mg/ml, and for cystatin C blots the samples were first diluted 1:25. The membranes were blocked in a solution containing 10% (w/v) milk proteins and probed with A11, polyclonal rabbit anti-human cystatin C antibodies (Dako) or the novel oligomer-specific antibodies. After incubation with HRP-labeled polyclonal swine antibodies against rabbit immunoglobulins, membranes were developed with ECL substrate and digitized in a ChemiDoc detection system.

##### Separate Measurement of Dimeric and Monomeric Cystatin C in Blood Plasma

To blood plasma from a healthy donor with a concentration of monomeric cystatin C of 0.9 mg/liter, was added stabilized dimeric cystatin C, isolated as described above, to a concentration of 1.7 mg/liter. To be able to separately measure monomeric and dimeric cystatin C after addition of oligomer-specific antibodies to the plasma, gel chromatography of the mixture on a column of Superdex 75 10/300 GL, run in an ÄKTA chromatography system, was used. The flow rate of the buffer (150 mm ammonium bicarbonate, pH 7.8) was 0.3 ml/min. The cystatin C concentration in the chromatography fractions containing monomeric or dimeric cystatin C was measured using the ELISA system as previously described (15).

## RESULTS

### 

#### 

##### Production of Stabilized Oligomers

**FIGURE 1.**
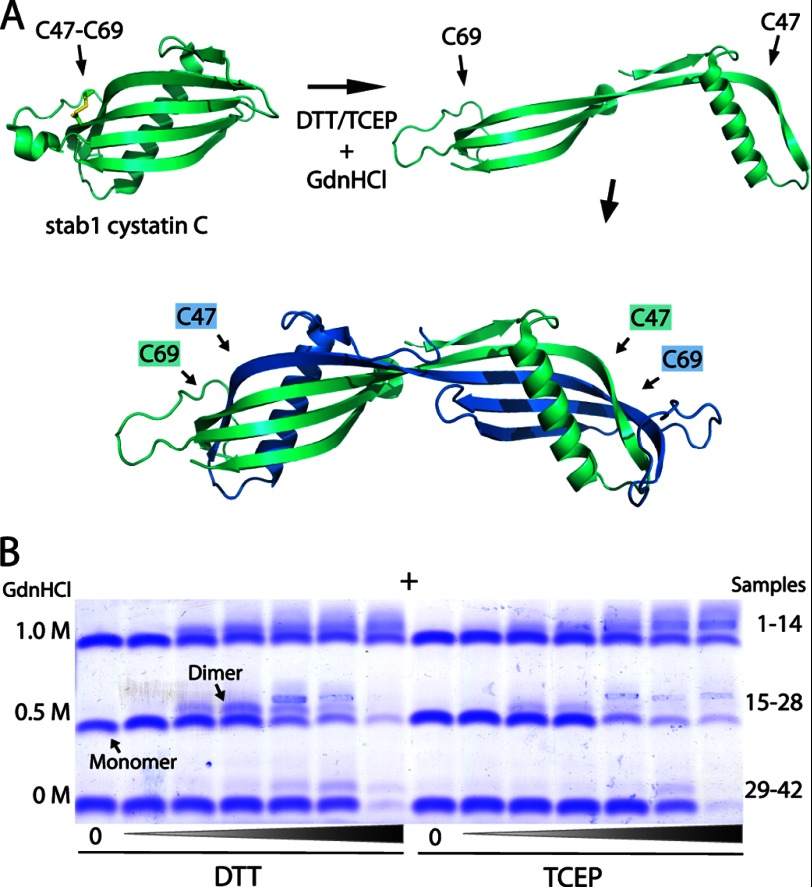
**Induced dimer formation of disulfide-stabilized cystatin C**. *A*, the crystal structure of monomeric L47C/G69C (denoted *stab1*) cystatin C is displayed in a *ribbon* representation together with partially unfolded and dimeric wt cystatin C, illustrating the mechanism of three-dimensional domain swapping whereby the first hairpin loop (L1) extends and tertiary structure elements are exchanged. The disease-associated L68Q variant shows rapid conversion to dimers under native conditions (17). Under nonreducing conditions stab1 cystatin C is stabilized by the added intramolecular disulfide bond and cannot undergo domain swapping and dimer formation. In a reduction reaction the disulfide is broken, allowing domain swapping and dimerization in mildly denaturing buffers. The figure was prepared in PyMol (43) using coordinates from the structures of Protein Data Bank numbers 3GAX (25) and 1TIJ (44). *B*, agarose gel electrophoresis of stab1 cystatin C samples, after incubation for 24 h at 40 °C in PBS, pH 7.4, in the absence (samples *1, 8, 15, 22, 29, 36*) or presence of the reducing agents DTT or tris(2-carboxyethyl)phosphine (*TCEP*) (samples *2–7, 16–21, 30–35,* and *9–14, 23–28, 37–42,* respectively) and low levels of GdnHCl. The optimum yield of conversion to dimers was obtained in 0.5 and 1.0 m GdnHCl by adding exactly 1:1 molar eq of DTT (samples *4* and *18*). Higher concentrations of reducing agents resulted in protein precipitation. The anode is marked by a *plus* sign.

We employed a miniaturized *in vitro* screening assay recently described (16) to find the optimal conditions for the induction of oligomer formation from monomeric stab1 cystatin C. As illustrated in Fig. 1*A*, this variant of cystatin C has two cysteines (L47C/G69C) introduced at strategic positions by site-directed mutagenesis, forming a disulfide link between the adjacent β2- and β3-strands, which (in wt cystatin C) separate during domain swapping and dimer formation (24). In the oxidized state the disulfide bond suppress this conformational flexibility of the monomer and prevents domain swapping (7). Our initial objective was to find near physiological conditions to induce domain swapping and dimer formation of monomeric stab1 cystatin C, by testing mildly denaturing parameters (heating, GdnHCl) combined with reducing agents (DTT and tris(2-carboxyethyl)phosphine) during different incubation time periods. In the optimal setting, the reducing agent should be perfectly titrated to leave the two intrinsic disulfide bonds (Cys^73^–Cys^83^, Cys^97^–Cys^117^) intact, and only reduce the additional (solvent-exposed) disulfide bridge to enable domain swapping of the monomeric protein. Indeed, after screening a number of different conditions, it was evident that when we used neutral buffers, pH 7.4, mild denaturation (0.5 or 1.0 m GdnHCl) and the addition of exactly 1:1 molar equivalents of DTT, we observed dimer formation without precipitation, as evident from agarose gel electrophoresis (Fig. 1*B*).

**FIGURE 2.**
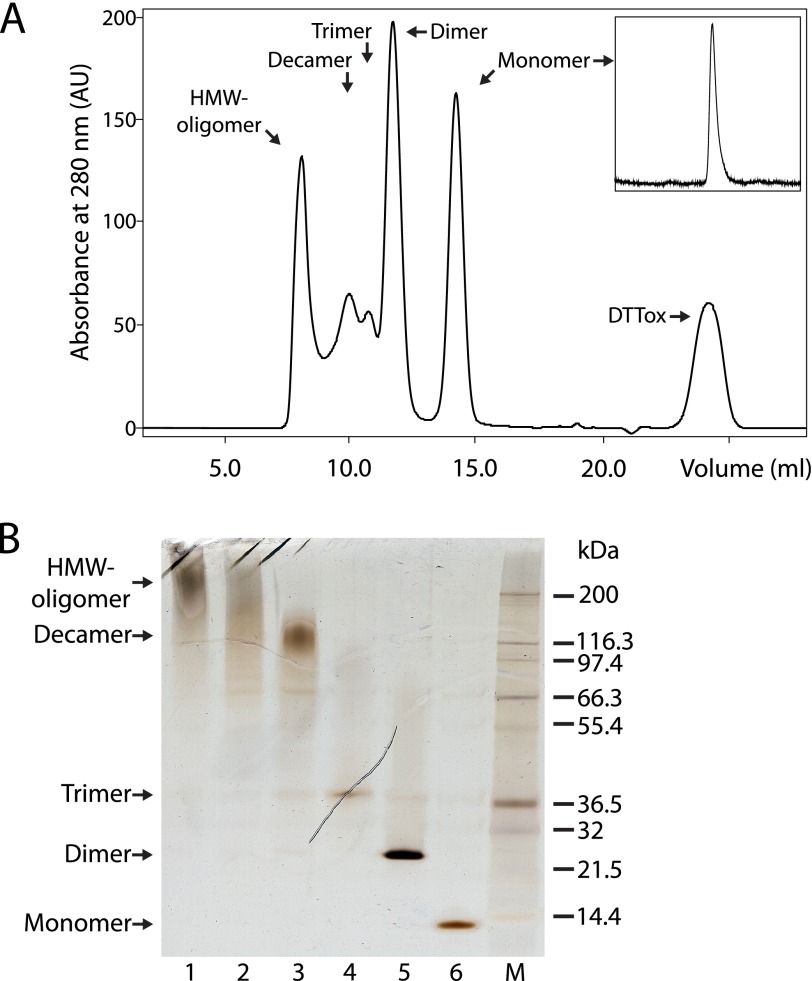
**Formation and purification of stabilized cystatin C oligomers.** *A*, when purified monomeric disulfide-stabilized (stab1) cystatin C (*inset*) was incubated at a high concentration (3 mg/ml) in PBS, pH 7.4, and 1 m GdnHCl at room temperature for 2 h with the addition of an equimolar amount of the reducing agent DTT, gel filtration, using a column of Superdex 75 GL 10/300, demonstrated the formation of several oligomeric species. A population of HMW-oligomers (>100 kDa) was eluted in the void volume and a peak representing oxidized DTT was observed (*DTTox*). *B*, SDS-PAGE displaying the stability and purity of cystatin C-containing fractions (*lane 1–6*) isolated by SEC. *M*, Mark12 protein ladder. Samples were boiled in 2% SDS without reducing agents prior to electrophoresis, and proteins were demonstrated by silver staining.

Next, analytical gel filtration (HPLC-SEC) was utilized to establish the optimal protocol for the induction of not only dimers, but also trimers and higher-order oligomers. This was accomplished by increasing the protein concentration, still retaining the 1:1 molar equivalents of DTT, and decreasing the temperature and incubation time to minimize protein loss by precipitation. By incubating mixtures of GdnHCl and DTT-treated stab1 cystatin C at 3 mg/ml in 0.5-ml volumes at room temperature, we were able to obtain and purify a number of cystatin C oligomers using preparative gel filtration (Fig. 2*A*). The yield of some oligomers was low, but, after ultrafiltration and re-chromatography, high enough to permit their characterization. Fractionation of the incubation mixtures by gel filtration in ammonium bicarbonate buffers enabled not only isolation of oligomers, but also removal of salts and oxidized DTT. All of the oligomers were SDS-stable (Fig. 2*B*), but DTT-sensitive, in SDS-PAGE (data not shown), indicating that intermolecular disulfide bridges stabilized them, thus supporting a model of domain swapping in the formation of oligomers (7, 21). Concentrated samples of the stabilized oligomers could be stored at −20 or 4 °C for more than 1 year, or pass through lyophilization and reconstitution cycles in aqueous buffers with pH values ranging from 4 to 9, without alteration in size, according to nonreducing SDS-PAGE.

##### Characterization of Stabilized Oligomers

To determine the molecular mass and number of monomeric subunits in each of the isolated oligomeric species, we used SDS-PAGE, mass spectrometry, native HPLC-SEC, transmission electron microscopy, and atomic force microscopy, producing the results summarized in Table 1. SDS-PAGE showed bands corresponding to purified and stabilized monomers, dimers, trimers, decamers, and HMW-oligomers (Fig. 2*B*). Tetramers were obtained in some preparative runs and observed in some decamer fractions (Fig. 6*C*). The HMW-oligomers migrated in SDS-PAGE as a diffuse band with a molecular mass of >200 kDa, similar to the annular oligomers formed from wt and L68Q cystatin C, as previously described (21). Mass spectrometry analysis using MALDI-TOF revealed monomers, dimers, trimers, and tetramers, but failed to identify the mass of the larger oligomers. All fractions except the HMW-oligomers eluted as distinct and uniform chromatographic peaks in SEC analyses. Using a calibrated column of Superdex 200, the mass distribution of the fractions corresponded to monomers, dimers, trimers, and tetramers. The HMW-oligomeric fraction eluted in the void volume of the column. In both preparative and analytical gel filtrations, the decamer fraction displayed a delayed elution profile in a retention volume corresponding to 4.8 monomeric subunits.

**TABLE 1:** **Molecular mass of stabilized cystatin C oligomers** The experimental masses were obtained from isolated disulfide-stabilized cystatin C oligomers by three different methods and are related to the theoretical mass of monomeric stab1 cystatin C (13.377 kDa). Mass data are given in units of kDa and the calculated number of monomeric subunits displayed within parentheses.

	SDS-PAGE	MALDI-TOF MS	HPLC-SEC	Theoretical mass
Monomer	11.9 (0.9)	13.379 (1.0)	10.2 (0.8)	13.377 (1.0)
Dimer	21.0 (1.6)	26.761 (2.0)	26.0 (1.9)	26.754 (2.0)
Trimer	34.3 (2.6)	40.096 (3.0)	40.6 (3.0)	40.131 (3.0)
Tetramer	49.7 (3.7)	53.455 (4.0)	50.6 (3.8)	53.308 (4.0)
Decamer	127 (9.5)	NA*^a^*	63.8 (4.8)	133.770 (10.0)
HMW-oligomer	>200 (>15)	NA	NA	NA

*^a^* NA, not applicable.

**FIGURE 3.**
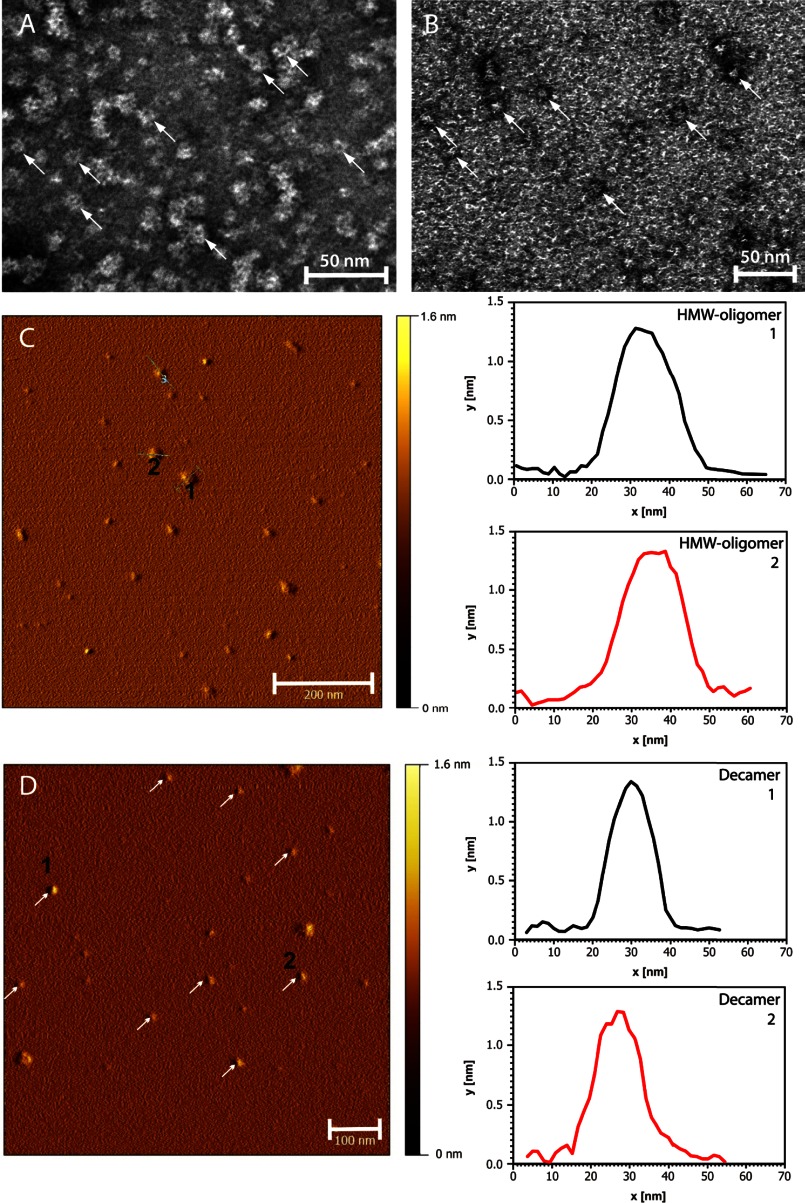
**Micrographs of stabilized cystatin C oligomers.** Negative-stain electron microscopy images of disulfide-stabilized HMW-oligomers (*A*) and decamers (*B*), isolated by SEC. In both samples circular structures were visualized (*arrows*), with some objects resembling the pore-like morphology of oligomers formed from several other precursor proteins (39–41). AFM images (*left*) were paired with duplicate diagrams (*right*) representing the cross-sectional analyses of HMW-oligomers (*C*) and decamers (*D*). The profiles of two HMW-oligomers and two decamers are given. The height of both oligomeric species is 1.2–1.4 nm, corresponding to a monomolecular layer of cystatin C subunits.

The HMW-oligomer and decamer fractions were examined by electron microscopy, and circular objects with most diameters in the 12–16 nm range were visible in the HMW-oligomer fraction (Fig. 3*A*). The decamer sample contained slightly smaller circular objects, with a diameter of 9–13 nm (Fig. 3*B*). A significant number of both the HMW-oligomeric and decameric objects displayed central cavities. The isolated HMW-oligomers and decamers were further characterized by AFM and, as shown in Fig. 3, *C* and *D*, the cross-sectional analysis showed a height of 1.2–1.4 nm for both objects. Crystallographic data for chicken cystatin and stab1 cystatin C indicate that this corresponds to monomolecular layers in both objects (25, 26). The resolution of the AFM tip used in the measurements did not allow visualization of the central cavities displayed by electron microscopy.

##### Biological Activity of Stabilized Oligomers

**FIGURE 4.**
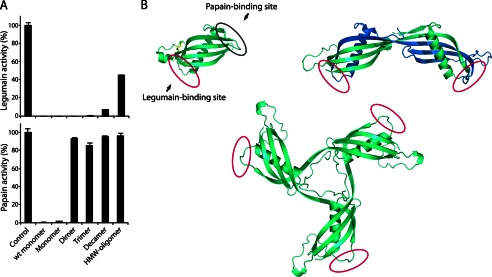
**Oligomers are domain-swapped and retain a native-like fold.** *A*, inhibitory capacity of isolated cystatin C oligomers tested against the two proteases, legumain and papain, both of which are inhibited by monomeric cystatin C. The *control* samples represent the full protease activity without inhibitors. Native, nonstabilized, monomeric cystatin C is denoted *wt monomer. Monomer*, *Dimer etc*. refer to disulfide-stabilized molecules. *Error bars*, S.D. *B*, crystal structure of monomeric stab1 cystatin C (Protein Data Bank entry 3GAX) (25) displaying the two protease binding sites residing on opposite sides of the molecule, where the N-terminal and two hairpin loops (L1 and L2) constitute the papain-binding domain and the legumain-binding domain resides in proximity to Asn^39^ (20). The crystal structure of dimeric cystatin C (Protein Data Bank entry 1TIJ) shows disruption of the papain-binding loop L1 that acts as a hinge to the swapping domain, whereas the fold of the legumain-binding domain is retained and accessible. The three-dimensional model of trimeric cystatin C was constructed in PyMol by repositioning the domain swapping structural elements (residues 1–57) in the monomer (Protein Data Bank entry 1TIJ) (44). The oligomer model is in agreement with previous experimental data obtained for dimeric wt cystatin C and the presented inhibitory profiles for stabilized oligomeric cystatin C.

To investigate the conformational changes induced during cystatin C oligomerization, we tested the inhibitory capacity of the stabilized oligomers toward the two cysteine proteases papain and legumain, both of which are inhibited by monomeric cystatin C (20). The stabilized dimer was as potent an inhibitor of legumain (102%) as the stabilized monomer (100%) or wt monomeric cystatin C (101%) (Fig. 4*A*). The retained inhibitory capacity toward legumain was also shown for the stabilized trimer (99%) and was only partially lost in the decamer and HMW-oligomer fractions (93 and 55%, respectively). In contrast, the dimer and the other oligomers showed complete loss of inhibitory capacity toward papain (Fig. 4*A*). Fig. 4*B* shows structural models explaining the loss of the papain inhibitory activity, whereas the legumain inhibitory activity is preserved.

##### Stabilized Oligomers Display General Oligomer Epitopes Detected by the A11 Antibody

**FIGURE 5.**
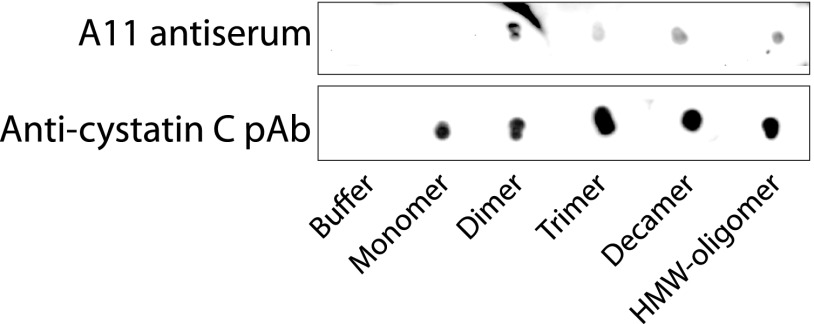
**Immunoblotting reveals common oligomeric epitopes in stabilized cystatin C oligomers.** Stabilized cystatin C oligomers and monomers, purified by SEC and concentrated by ultrafiltration, were dot-blotted onto nitrocellulose membranes and probed with A11 antibodies. A11 was originally raised against amyloid β-oligomers and reacts with oligomers of several other amyloidogenic proteins, but not with the protein monomers (27). In the control blot, samples were probed with commercially available polyclonal anti-cystatin C antibodies (*pAb*).

Amyloid oligomers might display unique epitopes common to all oligomers, but not present in monomers, and oligomers formed from a number of different amyloidogenic proteins can be detected with use of the A11 antibody (27). As shown in Fig. 5, all isolated cystatin C oligomers, but not monomers, were recognized by A11, although the reaction was weak. This indicates that these oligomers share epitopes unique to oligomers.

##### Propagated Domain Swapping Is Required in the Formation of HMW-oligomers and Fibrils

**FIGURE 6.**
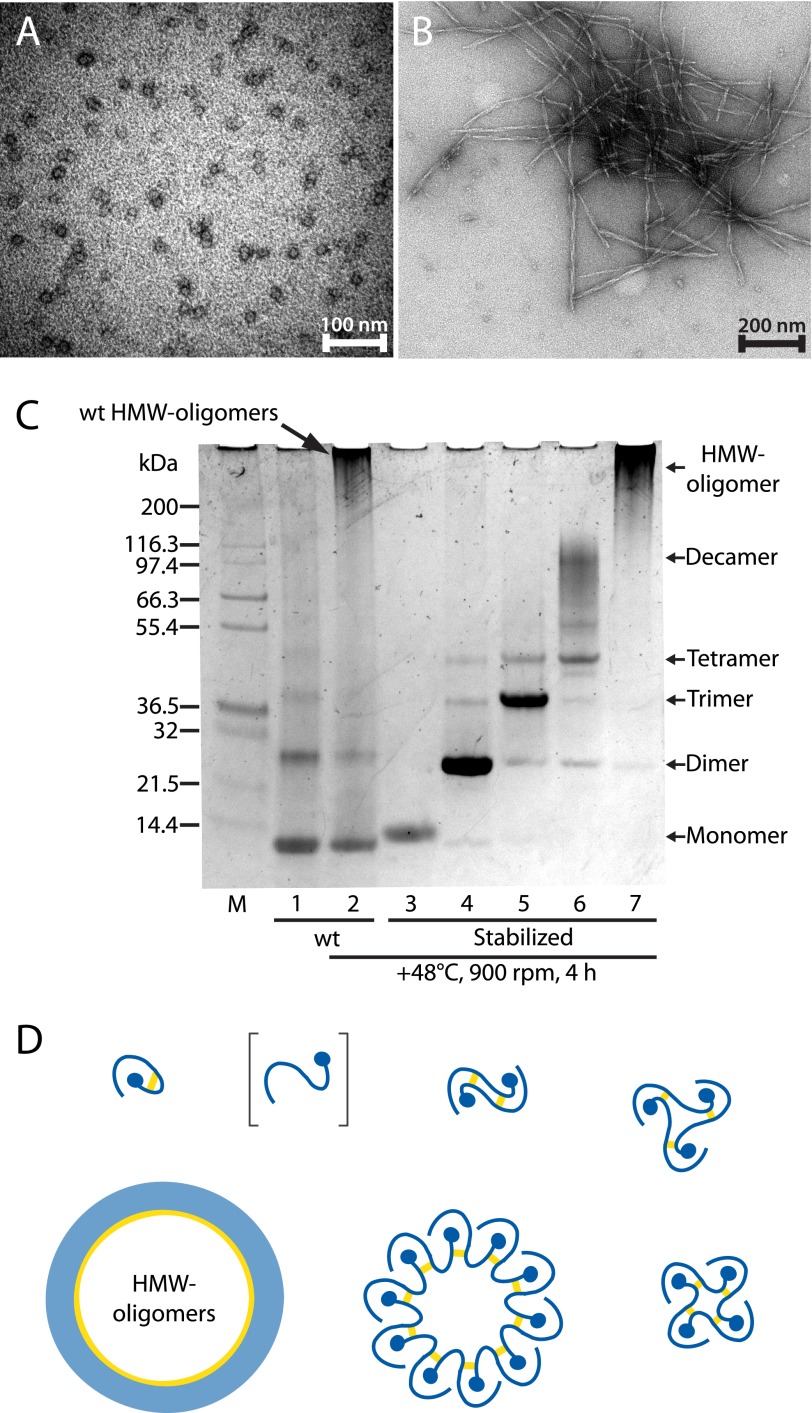
**Ring-closed stabilized oligomers are unable to propagate a domain swap.** *A*, electron micrograph of nonstabilized oligomers, produced by incubation at 48 °C for 4 h of wt cystatin C in 50 mm sodium acetate, pH 4.0, 100 mm NaCl. *B*, micrograph recorded after prolonged incubations of the sample in *A*, showing the formation of long twisted fibrils with approximate widths of 10 nm. *C*, SDS-PAGE run at 4 °C with nonreduced samples in 0.1% SDS to detect formation of HMW-oligomers. HMW-oligomers form after a 4-h incubation at 48 °C of monomeric wt cystatin C (*lane 2*) as detailed above, compared with the control sample stored at 4 °C (*lane 1*). Neither the disulfide-stabilized monomer (*lane 3*) nor the stabilized oligomers (*lane 4–7*) form any larger species after the incubation, suggesting that propagated domain swapping is required in the formation of larger oligomers. *M*, Mark12 protein ladder. *D*, model of the *in vitro* generation and molecular organization of cystatin C oligomers, where intermolecular disulfide bonds are re-formed in the isolated oligomeric species. The results in *C* show that the stabilized oligomers cannot oligomerize further, suggesting that no free thiols exist; *i.e.* all oligomers are formed by propagated domain swapping and closed head-to-tail.

To elucidate if smaller oligomers are merely building blocks in the formation of larger oligomers and fibrils, or if further oligomerization requires propagated domain swapping, we assessed the propensity for small stabilized oligomers to form HMW-oligomers. As reported previously (21) and shown for wt cystatin C in Fig. 6*A*, during the lag phase of fibril formation, already after 1 h of incubation at 48 °C in mildly denaturing buffers, pH 4.0, both wt and L68Q cystatin C form ring-shaped oligomers detectable by SDS-PAGE or electron microscopy. Prolonged incubation results in fibril formation, as shown for wt cystatin C after 3 weeks of incubation (Fig. 6*B*). When isolated stabilized cystatin C monomers, dimers, trimers, and decamers were incubated under these conditions, no formation of HMW-oligomers (Fig. 6*C*) or fibrils could be found. In contrast, nonstabilized monomeric wt cystatin C formed both HMW-oligomers and fibrils (Fig. 6, *A-C*). This supports the notion that domain swapping, propagated in an open-ended fashion, is a prerequisite for formation of, not only fibrils (7), but also of HMW-oligomers. A schematic model for the oligomerization process is given in Fig. 6*D*.

##### Production and Purification of Oligomer-specific Antibodies

**FIGURE 7.**
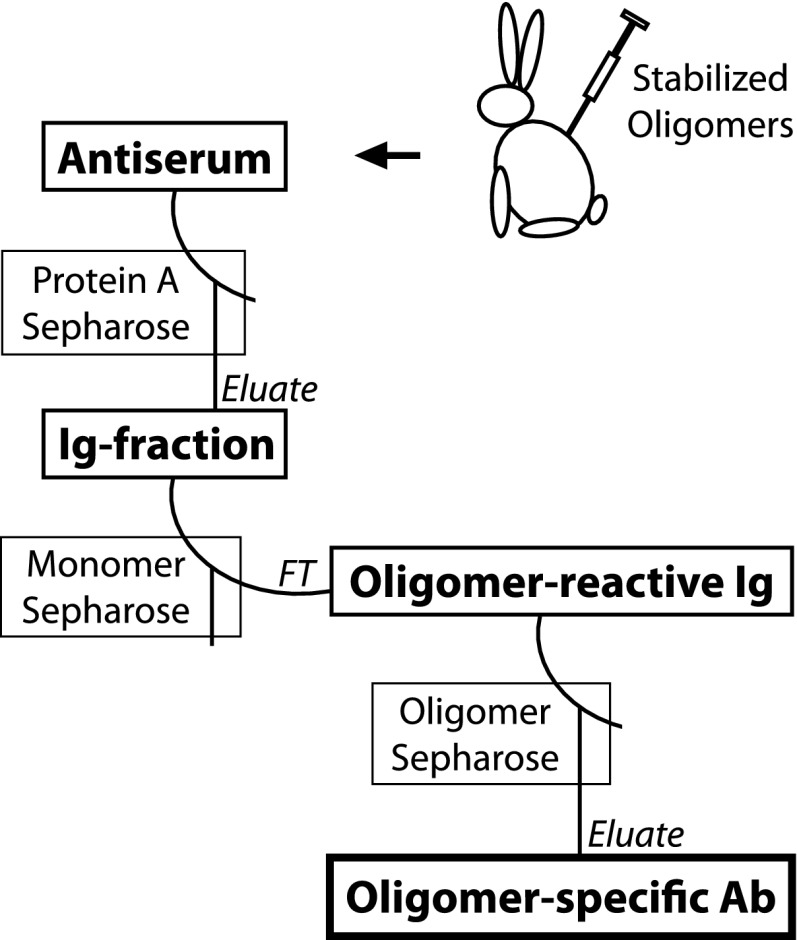
**Production and purification of oligomer-specific antibodies.** Schematic summary of the key steps in the isolation of oligomer-specific antibodies. Immunizations were performed using stabilized cystatin C dimers (0.1 mg/injection) or HMW-oligomers (0.05 mg/injection). The absorption of monomer-binding antibodies from the immunoglobulin fractions obtained was critical to avoid cross-reactivity in specificity assays (Fig. 8). *FT*, flow-through; *Ig*, immunoglobulin; *Ab*, antibodies.

**FIGURE 8.**
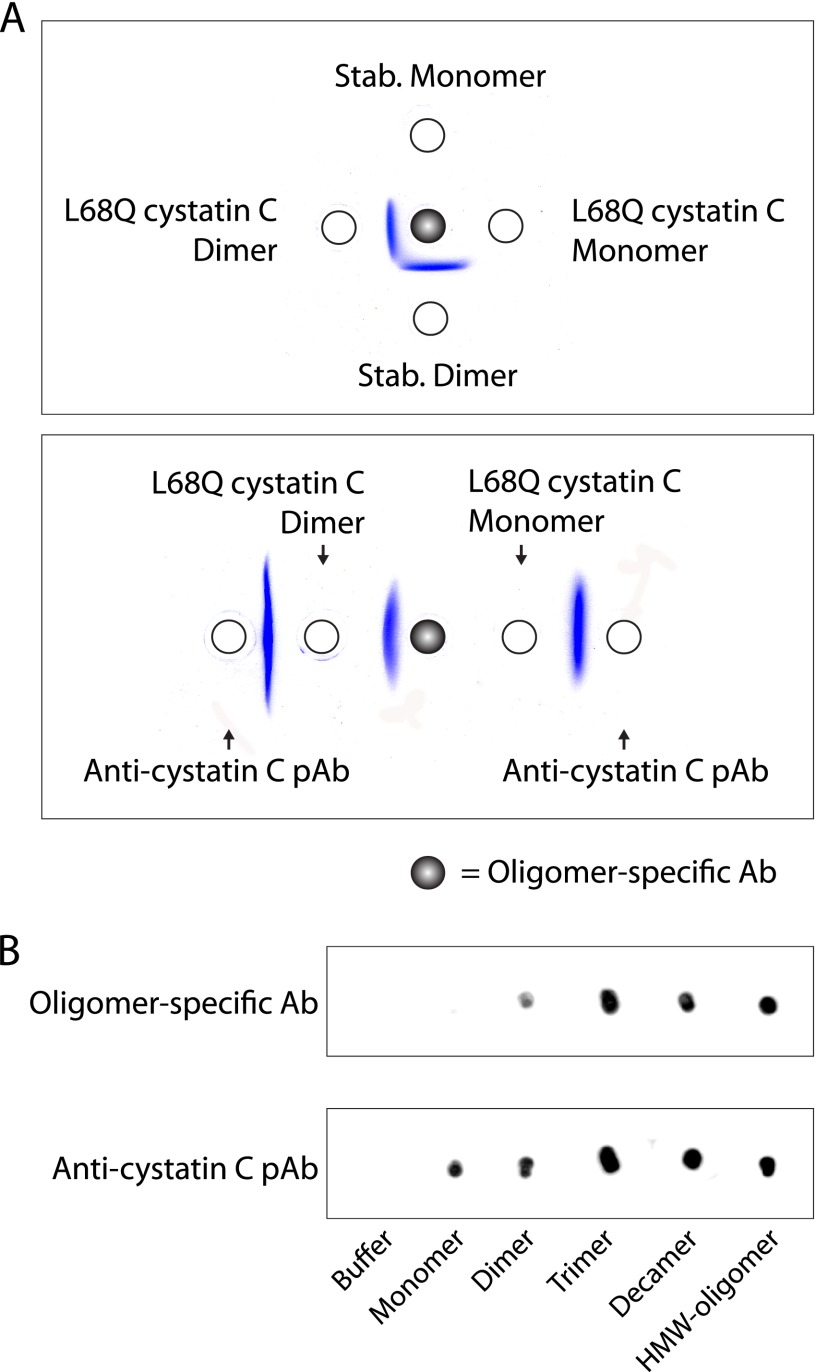
**Reactivity of oligomer-specific antibodies.** *A*, the specificity of antibodies, purified as described in the legend to Fig. 7 from the antisera obtained after immunization with stabilized cystatin C oligomers, was tested by double radial immunodiffusion. L68Q cystatin C dimers, associated with hereditary cystatin C amyloid angiopathy (42), showed antigenic identity with stabilized dimers, whereas monomers where nonreactive. Polyclonal antibodies raised against monomeric cystatin C (*anti-cystatin C pAb*) were used for comparison. *B*, immunoblots displaying the antigenic recognition of the oligomer-specific antibodies. Polyclonal antibodies raised against monomeric cystatin C (*anti-cystatin C pAb*) were used for comparison.

The successful purification and the evident stability of the isolated oligomers prompted us to immunize rabbits in an effort to raise antisera specific for oligomeric cystatin C. We chose the stabilized and isolated dimers and HMW-oligomers, respectively, as immunogens for immunization trials in two sets of rabbits, as described under “Experimental Procedures.” The specific antigen-antibody reactions of the antisera obtained were characterized by diffusion in-gel techniques. To obtain oligomer-specific antibodies without reactivity toward monomeric cystatin C, we purified the antibodies from dimer and HMW-oligomer immunized animals according to the scheme in Fig. 7. First, absorption of monomer-binding antibodies was accomplished by affinity chromatography of immunoglobulin fractions using Sepharose-coupled monomeric cystatin C. The flow-through fractions were collected, and the oligomer reactivity was verified (Fig. 8) before isolation of oligomer-specific antibodies using stabilized HMW-oligomers coupled to a Sepharose column. The fractions obtained by acidic elution from this column contained oligomer-specific antibodies from either dimer or HMW-oligomer-immunized rabbits.

##### Reactivity of Oligomer-specific Antibodies in Gel Diffusion and on Immunoblotting

The specific antibodies from oligomer-immunized rabbits precipitated stabilized dimers, but not monomers, in double radial immunodiffusion (Fig. 8*A*). They also precipitated stabilized HMW-oligomers (data not shown). No difference could be observed between antibodies obtained by immunizing with stabilized dimers and those obtained by immunizing with stabilized HMW-oligomers. The cerebral hemorrhage-causing L68Q variant of cystatin C spontaneously forms dimers (17) and the specific antibodies also precipitated these, but not monomeric L68Q cystatin C (Fig. 8*A*). The antibodies reacted strongly with HMW-oligomers, decamers, and trimers, and weakly with dimers, but not at all with monomers, in immunoblotting experiments (Fig. 8*B*).

##### Selectivity of Oligomer-specific Antibodies in Free Solution

**FIGURE 9.**
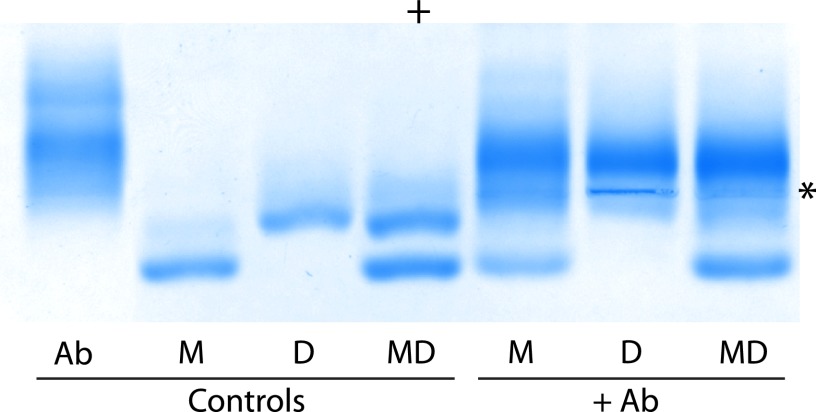
**Selectivity of oligomer-specific antibodies as shown by agarose gel electrophoresis.** The oligomer-specific antibodies were added to isolated monomers, dimers, or a mixture of monomers and dimers to assess their antigenic specificity. *Ab*, oligomer-specific antibodies; *M*, stabilized monomer; *D*, stabilized dimer; *MD*, mixture of stabilized monomers and dimers. An *asterisk* marks the point of the sample application and the anode is marked by a *plus sign*.

To assess the reactivity of the oligomer-specific antibodies in free solutions of dimeric and monomeric cystatin C, the following system was used. Mixtures of antibodies, stabilized monomers, and stabilized dimers were prepared and electrophoresed in agarose gels under native conditions. The results showed that dimers were the preferred antigen, although a minor monomer reactivity could be detected. As shown in Fig. 9, the antibodies could deplete a solution of dimeric cystatin C. Addition of the antibodies to a solution of both monomeric and dimeric cystatin C resulted in a shift of the dimer/monomer ratio from 60/40 in the control sample to 15/85, indicating the potential of these antibodies for selective removal of dimers and, probably, oligomers in general.

##### Oligomer-specific Antibodies Selectively Bind Dimers in Blood Plasma

**FIGURE 10.**
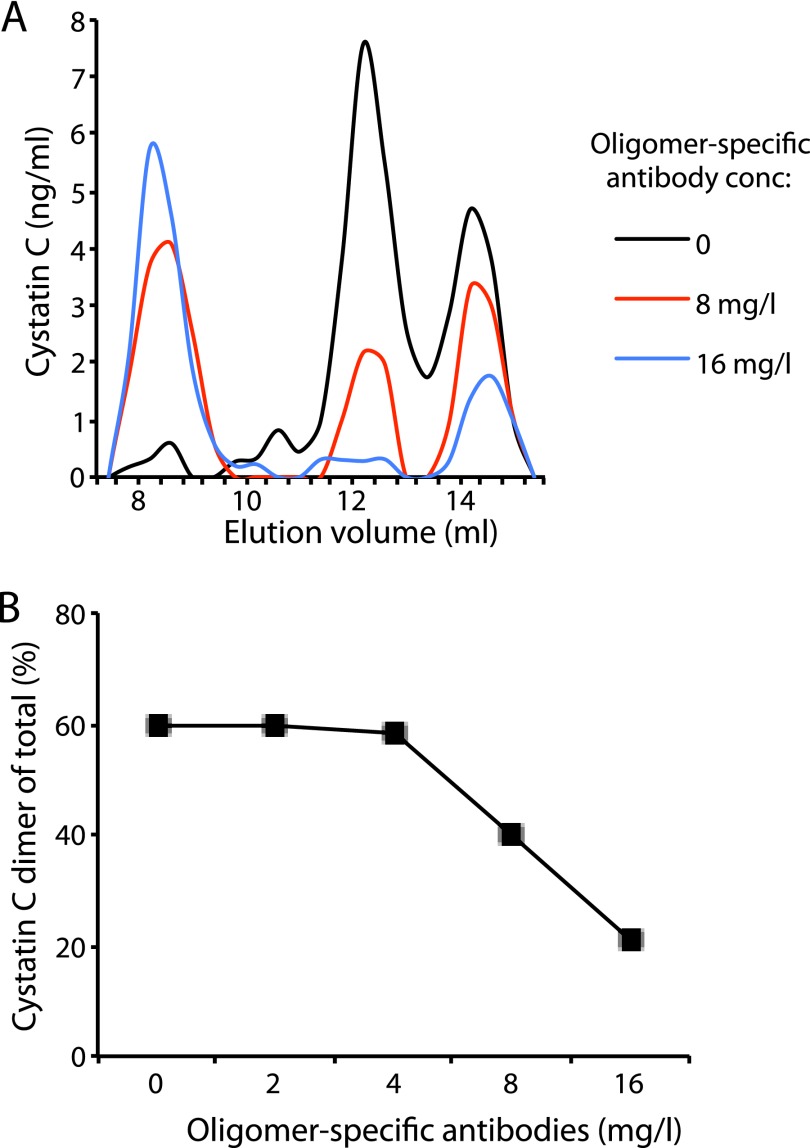
**Selective depletion of dimeric cystatin C in blood plasma.** Quantification of cystatin C in the fractions of gel filtration of plasma samples on a column of Superdex 75 GL 10/300 (*A* and *B*). In the control sample (*black line*) stabilized dimeric cystatin C was added to normal human plasma, resulting in a concentration of 1.7 mg/liter (endogenous monomeric cystatin C = 0.9 mg/liter). The oligomer-specific antibodies were added to the plasma sample 2 h before fractionation (*red* and *blue lines*). *B*, plot displaying the percentage of dimeric cystatin C of the total cystatin C (monomeric + dimeric) after the addition of increasing amounts of oligomer-specific antibodies.

To test if the oligomer-specific antibodies would selectively target oligomers also in complex biological systems, we developed a sensitive system for the detection of monomers and dimers in blood plasma using gel filtration followed by immunodetection (SEC-ELISA), as described under “Experimental Procedures.” The oligomer-specific antibodies were, in increasing concentrations, added to equal aliquots of plasma containing 0.9 mg/liter of monomeric and 1.7 mg/liter of dimeric cystatin C and, after a 2-h incubation at room temperature and centrifugation at 10,000 × *g* for 10 min, the mixtures were run in the SEC-ELISA system. A significant and dose-dependent decrease in cystatin C immunoreactivity in the fractions corresponding to dimers was observed, whereas monomeric cystatin C was less affected (Fig. 10). Using antibody concentrations of 8 and 16 mg/liter, respectively, corresponding to 50 and 100 nm, the dimeric cystatin C fraction was reduced by ∼65 and 95%, respectively (Fig. 10*A*). A concomitant increase in cystatin C immunoreactivity was also observed in the void volume of the column (SEC exclusion >100 kDa).

## DISCUSSION

Protein misfolding underlies neurodegeneration and chronic diseases commonly affecting the aging population. An important example is Alzheimer disease, which is characterized by progressive cognitive decline and cerebral amyloid deposition, and is expected to affect more than 100 million individuals by 2050 (28). No disease-modifying treatments are available (29), neither for Alzheimer disease nor for the other amyloid disorders, with rare exceptions (30).

In the amyloid disorders, a soluble functional monomeric protein is usually transformed into insoluble amyloid fibrils via several intermediary aggregates/oligomers, some of which display cytotoxic properties (4, 31). It has been suggested that a possible mechanism in this pathophysiological process is propagated domain swapping, in which continuous swapping of protein domains, present in the monomeric amyloidogenic proteins, produces the intermediary oligomers of different sizes and structures and, possibly, the final amyloid fibril (5, 7, 21, 32–36). Domain swapping has been demonstrated for a diverse set of amyloidogenic proteins, including cystatin C, β_2_-microglobulin, ribonuclease A, T7 endonuclease I, antithrombin, the prion protein, and amyloid β (5, 24, 32, 34–37). However, it is not clear if domain swapping is a general mechanism of amyloid aggregation, or if other mechanisms also exist. The structure and properties of the intermediary oligomers have been difficult to study due to their instability and dynamic equilibrium with smaller and larger species. This has hampered the development of treatments of amyloid disorders targeting such intermediary oligomers to reduce or eliminate their cytotoxicity or fibril producing capacity.

In hereditary cystatin C amyloid angiopathy (HCCAA), a mutation in the cystatin C gene produces a protein variant, L68Q cystatin C, which rapidly forms oligomers and amyloid deposition in the brain arteries, resulting in brain hemorrhage and death in early adult life (38). L68Q cystatin C forms oligomers and amyloid fibrils upon incubation *in vitro* under nondenaturing conditions, whereas wt cystatin C requires slightly denaturing conditions to produce oligomers and fibrils *in vitro* (7, 21). The production of oligomers and fibrils *in vitro* from wt cystatin C and L68Q cystatin C could be completely stopped by introducing a disulfide bridge in the proteins preventing domain swapping (7, 21). These protein variants were called stab1 cystatin C and stab1 L68Q cystatin C, respectively (7). By selective reduction of this disulfide bridge in stab1 cystatin C under slightly denaturing conditions *in vitro* and subsequent oxidation, low amounts of HMW-oligomers could be produced and investigated (21). In the present work, we have studied different *in vitro* conditions in an effort to produce, in addition to HMW-oligomers, other stable intermediary cystatin C-oligomers and partly characterize these. It was possible to find conditions allowing the production and isolation of several different stable cystatin C oligomers. Characterization of the isolated oligomers by SDS-PAGE, MALDI-TOF MS, HPLC-SEC, EM, and AFM indicated that they represented dimers, trimers, tetramers, decamers, and HMW-oligomers. The results concerning size, using SDS-PAGE, MALDI-TOF MS, and HPLC-SEC, agreed for the dimers, trimers, and tetramers (Table 1). But for the oligomers, which, as evaluated by SDS-PAGE, represented decamers, the HPLC-SEC results suggested a considerably smaller (pentameric) structure. However, EM and AFM of these oligomers showed circular objects, the size of which was more compatible with a decameric than a pentameric structure (Fig. 3). In addition to dimers, trimers, tetramers, and decamers, a more heterogenous fraction of HMW-oligomers were also produced. Interestingly, EM and AFM showed that both the decamers and the HMW-oligomers were round objects, although the size of the HMW-oligomers was larger and more variable than that of the decamers (Fig. 3). Furthermore, AFM indicated that the heights of both the decamers and the HMW-oligomers corresponded to a monomolecular layer of the subunits.

It is not known whether the specific set of oligomers (dimers, trimers, tetramers, and decamers) produced in our study are relevant to the set of oligomers present *in vivo* in different amyloid disorders. We have observed that the set is influenced by the incubation conditions. For example, the incubation conditions used for crystallization of monomeric cystatin C resulted in a population of nearly 100% cystatin C dimers (24). The present study indicates that the higher the concentration of monomeric cystatin C, the higher the amounts of larger oligomers formed. But the reason why virtually no pentamers, hexamers, heptamers, octamers, and nonamers are observed is not known. X-ray diffraction analysis of crystals of stabilized decamers, tetramers, and trimers, together with the known three-dimensional structure of dimeric cystatin C (24), might reveal if they share stabilizing structures, which are energetically unfavored in pentamers, hexamers, heptamers, octamers, and nonamers.

The homogeneity and stability of the oligomers might allow studies of their detailed structures using x-ray diffraction. This would be of particular interest for the decamers, as their gross structure, as defined by EM and AFM, is similar to those of oligomers described for other amyloidogenic proteins (39, 40). It is also noteworthy that a similar gross structure has been described for cytotoxic amyloid oligomers (41). A further indication that the decamer of cystatin C is similar in structure to oligomers of other amyloidogenic proteins is that the A11 antibodies, raised against amyloid β-oligomers and reacting with oligomers of several other amyloidogenic proteins (27), also react with the cystatin C decamers, but not with monomeric cystatin C (Fig. 5).

The stoichiometry of the reduction procedure used in the present study indicated that only the introduced disulfide bridge, preventing domain swapping in stab1 cystatin C, was reduced in the process, leaving the other two intramolecular disulfide bridges of native cystatin C intact (Fig. 1). Furthermore, an interesting property of the stable oligomers was that their inhibitory capacity for papain was completely lost, whereas their capacity to inhibit legumain was virtually unaltered, because each subunit of the oligomers inhibited approximately one legumain molecule. These properties are compatible with the known structures of monomeric cystatin C and its dimer. As displayed in Fig. 4*B*, the two inhibitory sites (for papain or legumain) of monomeric cystatin C are well separated. Our previous structure-function studies of dimeric cystatin C (20, 24) have shown that the papain-inhibitory site of monomeric cystatin C is completely disrupted during domain swapping and thus not present in dimeric cystatin C. In contrast, the legumain-inhibitory site is not influenced by the swapping of domains and thus intact in the cystatin C dimer (20). The results for the oligomers in the present study are therefore compatible with the hypothesis that a propagated domain swapping mechanism is operating in the formation of the oligomers, similar to the single domain swapping occurring in the formation of dimers (Fig. 4*B*). These results strongly suggest that the isolated oligomers are not random aggregates, but instead highly ordered, domain-swapped assemblies of monomeric cystatin C with the two disulfide bridges of native cystatin C intact in the subunits of the oligomers. The stability of the oligomers is most likely induced by novel intermolecular Cys^47^–Cys^69^ bonds, stabilizing the swapped domains.

One crucial part in understanding the pathophysiology of the amyloid disorders is to identify building blocks of HMW-oligomers and fibrils (4). The availability of stable small to medium sized oligomers might allow such studies. In the present investigation, mildly denaturing conditions, inducing the formation of HMW-oligomers from monomeric wt cystatin C, were used in an effort to produce such HMW-oligomers from stabilized monomeric, dimeric, trimeric, and decameric cystatin C. Surprisingly, no HMW-oligomers were produced from any of the smaller oligomers, which, as analyzed by SDS-PAGE, seemed to be unaffected by these incubation conditions (Fig. 6*C*). Therefore, at least for cystatin C, domain swapping seems to be required for all steps in the production of HMW-oligomers. This means that a substance, preventing (propagated) domain swapping, potentially would suppress the formation of larger oligomers from smaller ones and from monomeric cystatin C. It has previously been shown that formation of amyloid fibrils from monomeric wt cystatin C requires domain swapping (7).

Although the stabilized oligomers do not seem to be building blocks in the formation of amyloid fibrils, it will be of interest to investigate if any of them can catalyze the formation of amyloid fibrils from natural, nonstabilized, monomeric wt and L68Q cystatin C, as described for seeding of the incubation mixtures with sonicated preformed fibrils of wt and L68Q cystatin C (21).

The evident stability of the cystatin C oligomers also indicates that free cysteine thiols are absent, suggesting that all oligomers are closed by domain swapping head-to-tail (Fig. 6*D*). Such a mechanism, together with the monomolecular layer organization of subunits observed in AFM, might explain the toroidal (doughnut-shaped) morphology of the structures observed in EM and AFM. Similar morphologies have been described for the pore-like oligomeric assemblies described by others (39–41) and a similar mechanism of domain swapping head-to-tail might therefore be operating also in the formation of these assemblies. Importantly, pore-like oligomers might be the oligomeric structures responsible for cellular toxicity, by permeabilizing membranes and disrupting ion homeostasis (41).

The blood of patients with HCCAA contains dimers of L68Q cystatin C and perhaps larger oligomers (42). The availability of stabilized cystatin C oligomers raised the possibility of producing antisera specific for oligomeric cystatin C. Such antisera might be used as tools to reduce the amounts of cytotoxic oligomers and reduce the formation of HMW-oligomers and amyloid fibrils in HCCAA patients. They may also be used for diagnostic purposes, as the presence of cystatin C oligomers has been demonstrated only in HCCAA patients and not in healthy individuals (42). Indeed, immunization of rabbits with stabilized dimeric cystatin C, or stabilized cystatin C HMW-oligomers, and absorption of the antisera using columns of immobilized monomeric cystatin C, resulted in antisera with selectivity for oligomeric cystatin C, including cystatin C dimers. The best results were obtained when the specific antibodies were isolated by use of columns with immobilized stabilized HMW-oligomers. The specific antibodies could selectively precipitate, not only stabilized cystatin C dimers and oligomers, but also L68Q cystatin C dimers, present in HCCAA patients (Fig. 8). They could, in addition, be used to reduce the dimer-monomer cystatin C ratio, not only in pure solutions containing only these two molecular species, but also in complex biological fluids as blood plasma (Figs. 9 and 10). Interestingly, the immune response of all rabbits immunized with dimeric or HMW-oligomeric cystatin C were of an oligoclonal nature, as shown by the appearance of a few charge-homogeneous bands in the immunoglobulin zone of the agarose gel electropherograms of all antisera, exemplified in Fig. 9. This raises the possibility of producing rabbit monoclonal antibodies specific for oligomeric cystatin C, thus making generally available large pools of reagents with well defined properties for future research and treatment attempts. A further property of these antibodies, which might be interesting to study, is whether they, like the A11 antibodies, will react with amyloid oligomers of other proteins than cystatin C.

## Footnotes

2 The abbreviations used are:

MWCO: molecular weight cut-off
HMW: high molecular weight
AFM: atomic force microscopy
GdnHCl: guanidine hydrochloride
HCCAA: hereditary cystatin C amyloid angiopathy
SEC: size exclusion chromatography
BisTris: 2-[bis(2-hydroxyethyl)amino]-2-(hydroxymethyl)propane-1,3-diol.
